# The mechanosensor of mesenchymal stem cells: mechanosensitive channel or cytoskeleton?

**DOI:** 10.1186/s13287-016-0397-x

**Published:** 2016-09-20

**Authors:** E. Xiao, Chider Chen, Yi Zhang

**Affiliations:** 1 Department of Oral and Maxillofacial Surgery, Peking University, School and Hospital of Stomatology; National Engineering Laboratory for Digital and Material Technology of Stomatology, Beijing Key Laboratory of digital Stomatology, 22 Zhongguancun Nandajie, Haidian District Beijing, 100081 China; 2 Department of Anatomy and Cell Biology, University of Pennsylvania, School of Dental Medicine, 19104, Philadelphia, Pennsylvania USA

**Keywords:** Mesenchymal stem cells, Mechanosensation, TRPM7, Cytoskeleton

## Abstract

Mesenchymal stem cells (MSCs) are multipotent adult stem cells. MSCs and their potential for use in regenerative medicine have been investigated extensively. Recently, the mechanisms by which MSCs detect mechanical stimuli have been described in detail. As in other cell types, both mechanosensitive channels, such as transient receptor potential melastatin 7 (TRPM7), and the cytoskeleton, including actin and actomyosin, have been implicated in mechanosensation in MSCs. This review will focus on discussing the precise role of TRPM7 and the cytoskeleton in mechanosensation in MSCs.

## Background

Mesenchymal stem cells (MSCs) have been explored extensively in regenerative medicine due to their potential for differentiation into multiple tissue types. Recent studies have shown that both chemical and mechanical signals within the microenvironment direct MSC differentiation [1]. Mechanisms of MSC mechanosensation have been described. Putative mediators of MSC mechanosensation include transient receptor potential melastatin 7 (TRPM7), a mechanosensitive plasma membrane calcium channel, and the cytoskeleton [2–6]. Calcium mobilization has been well defined as a regulator of gene expression and cell behavior [7]. Both TRPM7 and the cytoskeleton were reported to be essential for mechanical stimulus-induced Ca^2+^ mobilization [2, 6] and subsequent differentiation [2, 5, 8]. The precise role of each subcellular component in mechanosensation and Ca^2+^ mobilization is, nevertheless, incompletely understood. In this article, we will focus on discussing current understanding of the membrane mechanosensitive channel and the cytoskeleton in the process of mechanosensation in MSCs.

### Mechanosensitive channels in MSCs

Mechanosensitive channels are widely reported as sensors of mechanical stimulation in multiple cell types, including epithelial cells, endothelial cells, and myocardial cells. Transient receptor potential (TRP), a calcium channel, including TRPV1, TRPV4, and TRPA1, are known to be involved in sensory signal transduction in a variety of species from *C. elegans* to higher vertebrates [9, 10]. In MSCs, there is accumulating evidence that mechanical stimulation regulates MSC behavior via Ca^2+^ mobilization [5]. We have characterized membrane TRPM7 in human bone marrow-derived MSCs serving as a mechanosensor and conducting calcium influx, which induced osteogenesis [2]. Adjacent to our work, two additional groups also implicated membrane TRPM7 in MSC Ca^2+^ influx in response to shear stress and stretch [5, 6].

**Fig. 1:**
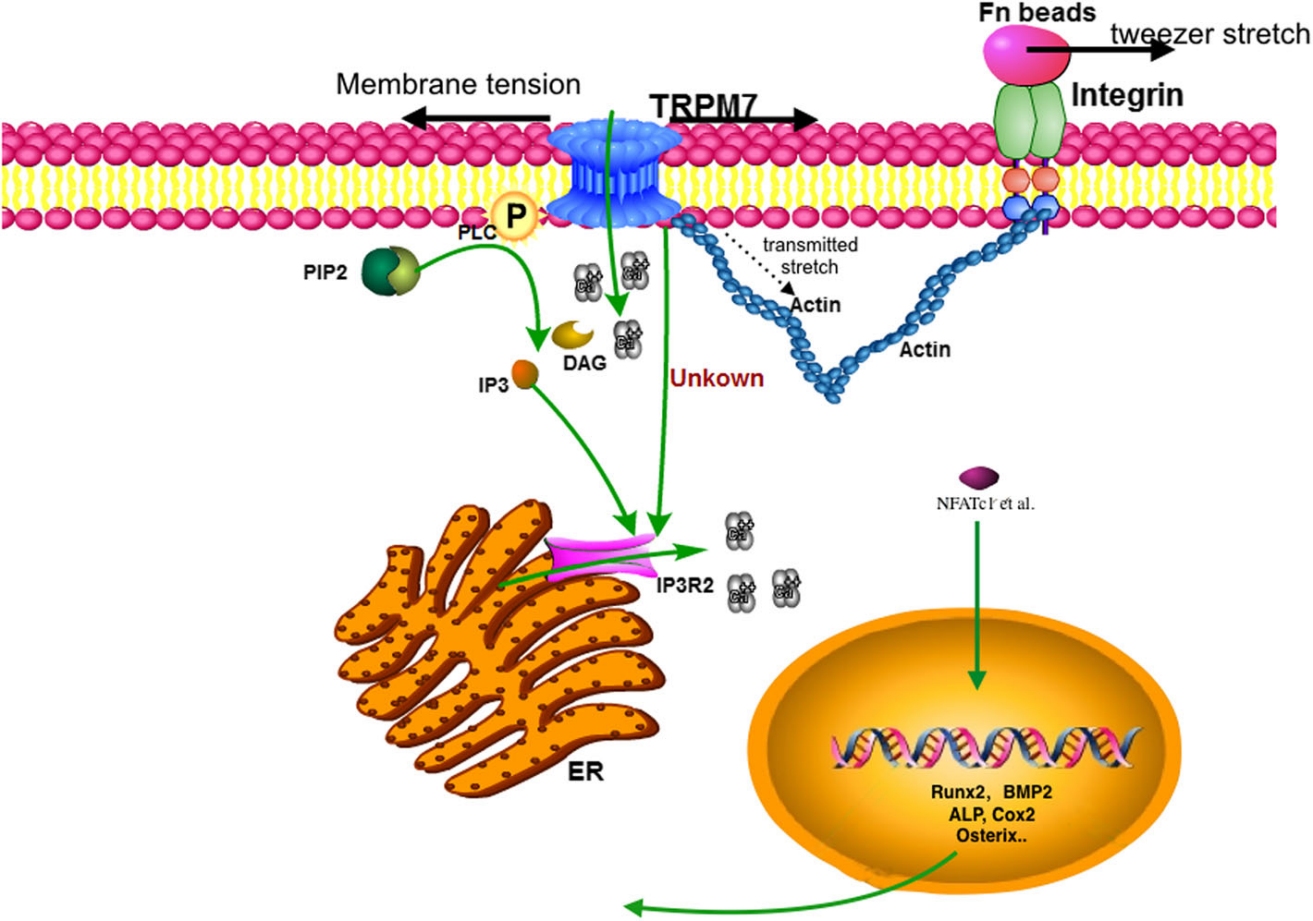
Two different models of the transient receptor potential melastatin 7 (*TRPM7*) mediation of mechanical stimulation in MSCs. *Left*: The bilayer lipid model. When mechanical stimulus is applied to the plasma membrane, TRPM7 is activated by membrane tension conducting Ca^2+^ influx. At the same time, cytophospholipase C (*PLC*) is activated and may hydrolyze phosphatidylinositol 4,5-bisphosphate (*PIP2*) to produce inositol trisphosphate (*IP3*) which subsequently activates inositol trisphosphate receptor type 2 (*IP*_*3*_ *R2*) on the endoplasmic reticulum (*ER*) conducting Ca^2+^ release. *Right*: The cytoskeleton tether model. When mechanical stimulus is applied to the cytoskeleton, it transmits stress that activates TRPM7-conducting Ca^2+^ influx, followed by activation of ER-conducting Ca^2+^ release. The exact linkage mechanism between TRPM7 and ER IP_3_Rs is still unknown. With mechanical stimulation, transcription factors like NFATc1 translocate to the nucleus and promote the osteogenic gene expression. *Alkaline phosphatase (ALP), Bone Morphogenetic Proteins (BMP), Diacylglycerols (DAG), Fibronectin (Fn)*

With mechanical stimuli, such as pressure, patch-clamp pipette suction, and patch-clamp pipette stretch, membrane TRPM7 opens and conducts Ca^2+^ influx from the extracellular space. Likewise, endoplasmic reticulum (ER) inositol trisphosphate receptor type 2 (IP_3_R2) Ca^2+^ release is also triggered by TRPM7 activation, which amplifies Ca^2+^ signaling. This increase in intracellular Ca^2+^ activates a downstream transcription factor, such as NFATc1, to induce osteogenesis [2]. TRPM7 activation appears to be independent of the cytoskeleton since disruption of actin polymerization by cytochalasin D does not abolish suction-induced TRPM7 activation [2]. Furthermore, membrane TRPM7 remains reactive to pipette suction in the absence of the cytoplasm as evidenced by patch clamp inside-out record model data [2]. TRPM7-regulated intracellular Ca^2+^ release, however, appears to be dependent upon inositol trisphosphate (IP3) since inhibition of cytophospholipase C (PLC), which hydrolyzes phosphatidylinositol 4,5-bisphosphate (PIP2) to produce IP3 upon interaction with TRPM7, abolished TRPM7-triggered ER Ca^2+^ release [2]. This model is illustrated in Fig. 1.

There are two general models that explain channel gating by mechanical stimuli. The bilayer lipid model proposes that force is delivered to the channel by surface tension or bending of the lipid bilayer causing a hydrophobic mismatch that favors channel opening. The tether model proposes that specific accessory proteins such as intracellular cytoskeletal elements or extracellular matrix molecules bind to channel proteins and transmit mechanical stimuli to the channel protein resulting in a channel conformational change and opening [10]. The data discussed above belong to the bilayer lipid model.

### The role of the cytoskeleton in mechanosensation in MSCs

The cortical cytoskeleton structurally supports the fluid bilayer which provides the cell membrane with shear rigidity, preserves cell deformability, and allows dramatic changes in cell shape and size. The actin cytoskeleton is a highly dynamic network which senses mechanical stimuli, remodels its own microstructures, and activates associated signaling pathways [11, 12].

In MSCs, actin skeleton, vinculin, and primary cilia are involved in mechanosensation [3, 4, 8, 13]. Primary cilia are made by the cytoskeleton component microtube and vinculin is a membrane-cytoskeletal protein in focal adhesion plaques that is involved in linkage of integrin adhesion molecules to the actin cytoskeleton. Both primary cilia and vinculin are co-localized with TRPM7 or other TRP channels. Kuo et al. reported that F-actin reorganizes in parallel with oscillatory shear stress and directs MSC differentiation by regulating β-catenin [3]. They propose that β-catenin coupling with integrin and cadherin on the membrane forms complexes with actin filaments, and that F-actin depolymerization releases these proteins to either activate downstream signaling pathways or be degraded. A study by Kim et al. further clarified the role of the cytoskeleton by directly applying stretch to integrin via fibronectin-coated beads and laser-tweezer [6]. Due to membrane reservoir compensation and direct linkage of integrin to actin, this model applies stimulus directly to the cytoskeleton rather than the plasma membrane [14, 15]. With this stimulus, membrane TRPM7 quickly activated and conducted extracellular Ca^2+^ influx (around 20 s after stimulation) faster than ER Ca^2+^ release (around 100 s after stimulation) [6]. Disruption of cytoskeletal actin by cytochalasin D, disruption of microtubules by nocodazole, or knockdown TRPM7 expression eliminated the force-induced Ca^2+^ oscillations. They found both activation of TRPM7 and ER Ca^2+^ release is dependent on the cytoskeleton (Fig. 1), while ER Ca^2+^ release upon mechanical stimulation was also depend on actomyosin contraction [6]. These results indicate that the cytoskeleton also directs response to mechanical stimulus, and that it can deliver mechanical force to membrane channels such as TRPM7 and the ER Ca^2+^ release channel.

### The coordinated cooperation of subcellular components in mechanosensation

According to current evidence, the membrane TRPM7 channel can be activated by either lipid bilayer tension or tethered cytoskeleton-transmitted mechanical stress to trigger ER IP3Rs Ca^2+^ release. Mechanosensation is a well-orchestrated process of three steps: mechanical transmission, signal activation, and signal amplification. When mechanical stimulus is applied to the plasma membrane or cytoskeleton, the lipid bilayer or cytoskeleton transmits the mechanical force to the mechanically gated Ca^2+^ channel TRPM7, resulting in Ca^2+^ influx. TRPM7 activation also triggers ER IP_3_R2 Ca^2+^ release via PLC-derived IP3 [2] or, potentially, by other mechanisms [6]. During this process, the transmission apparatus (lipid bilayer or cytoskeleton) triggers signal effector (mechanosensitive channel TRPM7) and signal amplifiers (ER IP_3_Rs), which work together as a system to transfer mechanical signals into biological responses. ’The data discussed above suggest that MSCs are unable to respond to mechanical stimulation in the absence of these elements.

## Conclusion

TRPM7 appears to play an important role in mechanosensation in MSCs. Since the transmission apparatus can be lipid bilayer or cytoskeleton. And the linkage between TRPM7 and ER IP_3_Rs can be PLC or another unknown mechanism. But the TRPM7 is indispensable for mechanical stimulus-activated Ca^2+^ influx and ER Ca^2+^ release and its downstream biological effects [2, 5, 6].

## Abbreviations

ER: Endoplasmic reticulum
IP3: Inositol trisphosphate
IP_3_R2: Inositol trisphosphate receptor type 2
MSC: Mesenchymal stem cell
PIP2: Phosphatidylinositol 4,5-bisphosphate
PLC: Cytophospholipase C
TRP: Transient receptor potential
TRPM7: Transient receptor potential melastatin 7
